# Antimicrobial Evaluation of Sulfonamides after Coupling with Thienopyrimidine Coplanar Structure

**DOI:** 10.3390/ph17020188

**Published:** 2024-01-31

**Authors:** Elshaymaa I. Elmongy, Wejdan S. Alanazi, Alhanouf I. Aldawsari, Asma A. Alfaouri, Reem Binsuwaidan

**Affiliations:** 1 Department of Pharmaceutical Chemistry, Faculty of Pharmacy, Helwan University, Ain Helwan, Cairo P.O. Box 11795, Egypt; shaymaa.taha@pharm.helwan.edu.eg; 2 College of Pharmacy, Princess Nourah bint Abdulrahman University, P.O. Box 84428, Riyadh 11671, Saudi Arabia; wejdan.saad18@gmail.com (W.S.A.); hanouff568@gmail.com (A.I.A.); asma.ayed.al@gmail.com (A.A.A.); 3 Department of Pharmaceutical Sciences, College of Pharmacy, Princess Nourah bint Abdulrahman University, P.O. Box 84428, Riyadh 11671, Saudi Arabia

**Keywords:** thienopyrimidine–sulfonamide hybrids, antimicrobial activity, heterocyclic compounds

## Abstract

This work describes the design and synthesis of three series of hybrids of thienopyrimidines and sulfonamides. Dihydrofolate reductase enzyme was selected as a target for the in-silico screening of the synthesized thienopyrimidine–sulfonamide hybrid as an antibacterial, while squalene epoxidase was selected as an antifungal target protein. All screened compounds showed promising binding affinity ranges, with perfect fitting not exceeding 1.9 Å. The synthesized compounds were tested for their antimicrobial activity using agar well diffusion and minimum inhibitory concentration tests against six bacterial strains in addition to two *Candida* strains. Compounds **8iii** and **12ii** showed varying degrees of inhibition against *Staphylococcus aureus* and *Escherichia coli* bacterial strains, whereas the best antifungal activity against *Candida* was displayed by compound **8iii**. Compound **12ii**, the cyclohexathienopyrimidine coupled with sulfadiazine at position 3, has the best antibacterial activity, which is consistent with molecular docking results at the active site of the oxidoreductase protein. Interestingly, compound **12ii** also has the highest docking binding energy at the antifungal squalene epoxidase active site. Investigating the physicochemical properties of the synthesized hybrids revealed their high tolerability with cell membranes, and moderate to poor oral bioavailability, and that all are drug-like candidates, among which **4i**, the cyclohexathieno[2,3-*d*] pyrimidine core with sulphaguanidine incorporated at position 4, recorded the best score (1.58).

## 1. Introduction

Scientific research is participating in significant advancements in diagnosis, prevention, and therapy. Recent studies have shifted their focus to heterocyclic compounds, especially those containing a thienopyrimidine core. These compounds have garnered attention due to their unique structural similarity to purines and their versatile pharmacological properties. They have been shown to possess antimicrobial [1], antiviral [2], and anti-inflammatory [3,4] properties, serve as β3-adrenoceptor agonists [5], exhibit anti-tuberculosis properties [6], antiprotozoal activity [7], and kinase inhibition [8,9,10,11,12], act as antioxidants [13,14,15,16], and possess anticancer activity [17,18,19,20].

A concerning global issue is the growing prevalence of pathogenic microorganism infections caused by bacteria such as *Staphylococcus aureus* and *Escherichia coli*, and the fungal strain *Candida albicans*. These strains have demonstrated resistance to commonly used antibiotics [21,22]. Therefore, there is an urgent need to develop novel molecules as antimicrobial agents. Recent studies on derivatives of thieno[2,3-*d*]pyrimidine have revealed their effectiveness against both gram-negative and gram-positive bacteria [22,23,24,25], making them promising candidates for the development of a new class of antibacterials. The molecular structure of thienopyrimidine compounds contains features necessary to interact with microbial targets, resulting in antimicrobial activity [26]. The arrangement of rings, functional groups, and bio-isosteric replacements can be adjusted to enhance potency, selectivity, and safety, making thienopyrimidines promising building blocks for the development of new antimicrobial agents [27]. 

Sulfonamides have diverse biological activities including bacterial [28,29] and protozoal [30,31] activities, as well as acting as dihydropteroate synthase inhibitors, thus inhibiting the biosynthesis of dihydrofolic acid [32], inhibiting carbonic anhydrase (CA) [33] and epidermal growth factor receptor (EGFR) [34,35], and inducing insulin release [36], as well as having antiviral [37], antifungal [38], anticancer [39,40], and anti-inflammatory activities [41]. 

### Design Strategy

Scientific groups have reported on the successful implementation of the hybrid pharmacophore concept, which is performed through combining heterocycles with recognized active groups like sulfonamides [42,43]. Hybridized heterocycles with sulfonamides were successfully applied in the reported literature as seen in the interconnection between phthalazinone derivatives and sulfonamides as sulfadiazine and sulfathiazole, which was a successful strategy to synthesize broad-spectrum antibacterial compounds [28]. Also, hydrophilic ends as guanidine in sulfaguanidine are present in the residue arginine (Arg), which has been noticed in many protein binding sites. In addition, it forms an important therapeutic agent when it is incorporated in the structure due to its reported biological effects, especially in antibiotics such as trimethoprim [44]. Additionally, the thienopyrimidine heterocyclic structure has shown promising antimicrobial activity when combined with sulfa compounds. Their combination has been explored due to their potential to combat various microbial infections [44,45].

Accordingly, the design strategy of this study was based on the introduction of the cyclohexylthieno[2,3-*d*]pyrimidine heterocyclic as a core structure, as thieno[2,3-*d*]pyrimidine has been previously reported as a promising scaffold for antimicrobial compounds, followed by the incorporation of different sulfonamide derivatives, given that they are an important class of antibiotic drugs with a wide range of activity, led by compounds **I**–**III** [46,47]. Thus, our research focused on combining thienopyrimidine’s coplanar cyclic structure with various substituted sulfonamide groups, which were initially incorporated at position 4 of the thienopyrimidine core. A series of thienopyrimidine–sulfonamide hybrids, which we have designated as “**4i**–**iii**”, were synthesized, one of which—compound **4ii**—exhibited mild antibacterial activity. The sulfonamides sulfadiazine and sulfamethoxazole were selected as references for comparison. Aiming to further explore the activity, the substitution was then shifted from position 4 to position 3 to synthesize the novel series “**12i**–**iii**”. The thienopyrimidine–sulfadiazine hybrid **12ii** demonstrated enhanced antibacterial activity. 

**Figure 1:**
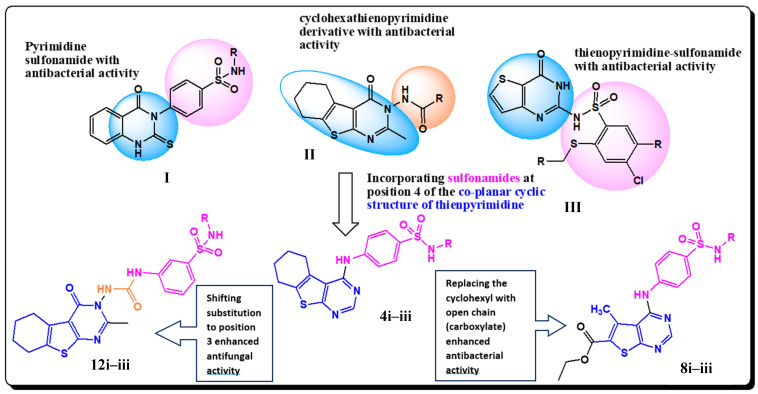
Design strategy for the synthesized theinopyrimidine–sulfonamide hybrids series **4i**–**iii**, **8i**–**iii**, and **12i**–**iii**.

In an attempt to further explore the hybrids’ activities, we investigated the effect of replacing the cyclic cycloalkyl ring with a carboxylate open chain, resulting in series “**8i**–**iii**”, which revealed enhanced antifungal activity. The design strategy is illustrated below (Figure 1).

## 2. Results and Discussion

### 2.1. Molecular Modeling Studies

Molecular docking studies were performed on the prepared compounds **4i**–**iii**, **8i**–**iii**, and **12i**–**iii**. The study of the prepared structures was undertaken on an antibacterial target protein (PDB ID: 2W9S) and an antifungal target protein (PDB ID: 2AIB), whose crystal structures bound to their co-crystallized ligands were downloaded from the Protein Data Bank. 

**Figure 2:**
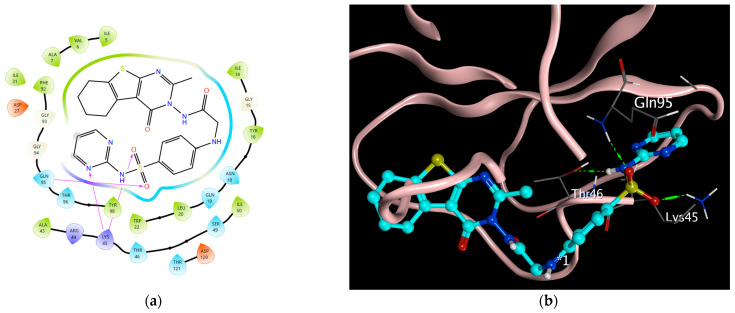
Types of bonding between the compound with the best docking score as well as antibacterial biological results (compound **12ii**; cyan balls and sticks) and the amino acid residues at the binding pocket of the protein with PBD ID 2W9S; H-bonds are represented by green dotted lines. *1 is the position of the co-crystalized ligand “trimethoprim”. (**a**) 2D structure; (**b**) 3D illustration.

Chemotherapeutic chemicals such as antibiotics are used to either suppress or kill germs. Sulfonamides are structural analogues and competitive antagonists of p-aminobenzoic acid in the manufacture of folic acid, which is necessary for bacteria to continue producing DNA. Tetrahydrofolate synthesis is inhibited by sulfonamide medications in conjunction with trimethoprim, further impeding DNA replication. The drug’s effects cause obstacles to cell division [48]. Accordingly, the dihydrofolate reductase (DHFR) enzyme was selected as a target for in-silico screening using molecular docking to compare the interactions of the newly synthesized thienopyrimidine–sulfonamide hybrids with those of the co-crystallized ligand (trimethoprim). The protein with PDB code 2W9S was downloaded and complexed with trimethoprim. All the screened compounds showed promising binding affinities ranging from −8.7115 to −7.1696, with perfect fitting not exceeding 1.9 Å (Table 1). The best binding affinity (−8.7115) was recorded by carboxylate thieno[2,3-*d*]pyrimidin-3(4*H*)-yl)-2-((4-(*N-*(pyrimidin-2-yl)sulfamoyl)phenyl)amino) acetamide derivative **12ii**, with a root mean square deviation (RMSD) value of 1.3166 Å. The interactions with the active site occurred at three positions with the amino acid residues THR 46, LYS 45, and GLN 95. These interactions involved a hydrogen bond interaction between the NH group of compound **12ii** and the acceptor oxygen of THR46, and two hydrogen bonds between the donor nitrogen atoms of both LYS 45 and GLN 95 and the acceptor sulfoxide group of thienopyrimidine–sulfadiazine-synthesized hybrid **12ii**, (Figure 2). Interestingly, the compound **12ii** had the best docking results as well as the best antibacterial results among the screened compounds, demonstrating enhanced antibacterial activity better than sulfadiazine alone against gram-positive *S. aureus*.

**Figure 3:**
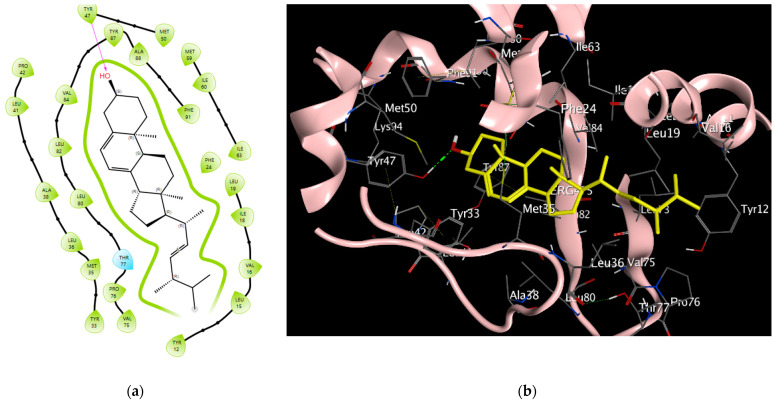
Ergosterol, the co-crystallized ligand of protein PDB ID 2AIB, interacting at TYR 47 of the receptor pocket; (**a**) 2D structure; (**b**) 3D illustration.

Moreover, squalene epoxidase (SE), cytochrome P450 sterol 14a demethylase (CYP51), and β-1,3-glucansynthase are the primary targets of antifungal screening [49]. In this work, antifungal activity was investigated using molecular docking on SE. Ergosterol production involves squalene epoxidase, and, therefore, inhibitors target this domain [50]. Ergosterol has a coplanar four-cyclic structure that resembles the core structure under investigation. The structure of the target protein with its co-crystalized ligand (PDB ID: 2AIB) was downloaded and showed that the main key interaction involves the TYR 47 amino acid residue (Figure 3). The docking results of the thienopyrimidine–sulfonamide hybrid series **4**, **8**, and **12** upon docking at the active site, taking the co-crystallized ligand as a placement guide, were very promising, with binding energies ranging from −6.807 kcal/mol to −9.6592 kcal/mol. 

**Figure 4:**
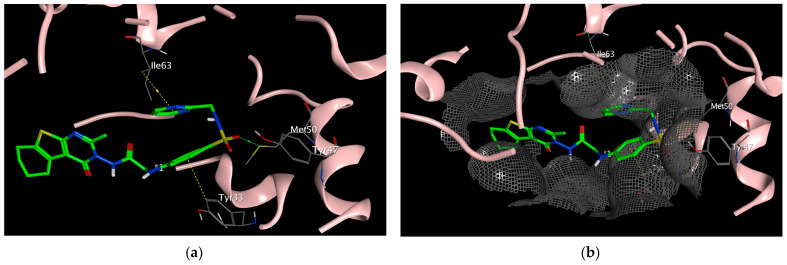
The best docking results for antifungal screening (compound **12ii** presents as green sticks); (**a**) 3D structure with the residues involved in the interaction where hydrogen bonds are green dotted lines and hydrophobic interactions are yellow dotted lines; (**b**) 3D illustration of **12ii** totally included in the binding site of action (represented by grey net).

**Figure 5:**
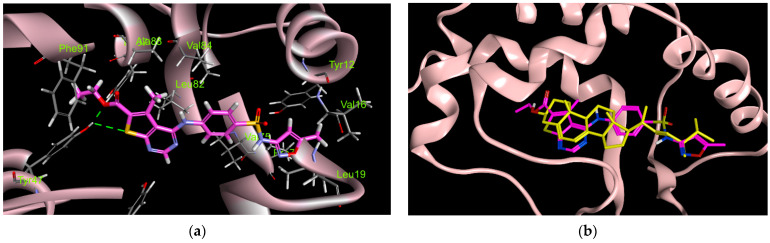
The best compound in antifungal biological results (compound **8iii** presents as pink sticks); (**a**) 3D structure with the residues involved in the interaction where hydrogen bonds are green dotted lines; (**b**) 3D overlay complex between compound **8iii** and ergosterol (the co-crystallized ligand represented in yellow sticks) at the pocket of 2AIB protein.

The thienopyrimidine–sulfadiazine hybrid **12ii** had the best docking score, with a binding energy of −9.3391 kcal/mol and a perfect fit at the site of interaction with an RMSD value of 1.7258 Å, (Figure 4). Compound **8iii** had the best antifungal results, forming two hydrogen bond interactions at −8.2032 kcal/mol with an RMSD of 1.8383 Å with the active site at TYR47, which is a promising biological result. TYR 47, THR 74, MET 50, and VAL 75 were the main receptor residues involved in most interactions between the synthesized ligands and the active site, (Figure 5). However, it is worth noting that compound **12ii**—the cyclohexathienopyrimidine coupled with sulfadiazine at position 3—exhibited the best antibacterial activity, which is consistent with its molecular docking results at the active site of the DHFR oxidoreductase protein. Interestingly, **12ii** also had the highest docking binding energy at the antifungal squalene epoxidase active site. 

Table 1 and Table 2 present the docking results of the synthesized hybrids, which include binding affinity scores and RMSD values as well as the ligand interactions (hydrogen bonding or hydrophobic interactions) with the active site residues.

### 2.2. Antimicrobial Investigation

Thienopyrimidine compounds have demonstrated significant antimicrobial activity, making them a promising avenue in the search for novel antimicrobial agents [25]. One of the key factors that contributes to the antimicrobial activity of thienopyrimidine compounds is their ability to disrupt essential cellular processes in microorganisms. Thienopyrimidine compounds can be tailored to act selectively against specific microbial targets. For example, some thienopyrimidines have been shown to selectively inhibit key enzymes involved in bacterial DNA replication, transcription, translation, and cell wall synthesis [44]. Through targeting essential microbial processes, these compounds disrupt vital cellular functions, leading to the inhibition or killing of different microorganisms [25]. Furthermore, thienopyrimidines have also demonstrated effective antifungal activity [1,27,51]. The unique structures of these compounds allow them to target specific fungal enzymes, which leads to compromised cell membrane integrity and, consequently, fungal cell death. 

In this study, three series of thieno[2,3-*d*]pyrimidine derivatives (**4i**–**iii**, **8i**–**iii**, and **12i**–**iii)** were investigated for their antimicrobial activity against different bacterial and fungal strains. The synthesized compounds were tested for their antimicrobial activity using agar well diffusion and MIC tests using serial dilution against the following bacterial and fungal strains: *Staphylococcus aureus* (ATCC 25923), *Staphylococcus aureus* (ATCC 29213), *Staphylococcus epidermidis* (ATCC 12228), *Enterococcus faecalis* (ATCC 29212), *Escherichia coli* (ATCC 25922), *Pseudomonas aeruginosa* (ATCC 27853), *Klebsiella pneumoniae* (ATCC 700603), *Candida albicans* (ATCC 10231), and *Candida parapsilosis* (ATCC 22019). The results were expressed as the average diameter of the growth inhibition zone (GIZ) and the minimum inhibitory concentration (MIC).

Five of the investigated compounds showed activity against the gram-positive strain *Staphylococcus aureus* but not against *Staphylococcus epidermidis* and *Enterococcus faecalis.* Among the three tested gram-negative strains, five of the investigated thienopyrimidine–sulfonamide hybrids showed mild activity against *Escherichia coli* but with smaller zones of inhibition and higher MIC values than sulfonamides alone. No activity was detected against *Pseudomonas aeruginosa* nor *Klebsiella pneumoniae,* Table 3.

Regarding antifungal activity, six of the tested hybrids displayed good activity against the tested *Candida* strains, as tabulated in Table 4. Five of the investigated compounds—**4ii**, **8ii**, **8iii**, **12i**, and **12iii**—showed comparable or better activity than the investigated sulfonamides in both zones of inhibition and MICs. 

### 2.3. Correlating Structure to Biological Activity

Linking the coplanar cyclic structure of thienopyrimidine with different substituted sulfonamide groups was initially performed at position 4 of the thienopyrimidine core. This afforded a series of thienopyrimidine–sulfonamide hybrids designated as “**4i**–**iii**”, among which the cyclohexathienopyrimidine–sulfadiazine hybrid **4ii** exhibited mild antibacterial activity with zones of inhibition of 15 mm for *S. aureus* gram-positive bacteria and 18 mm for *E. coli* gram-negative bacteria in comparison to both references, sulfadiazine and sulfamethoxazole, which recorded zones of inhibition of 29.67 mm and 27.67 mm, respectively. In addition, its antifungal activity was the best among series 4, reflected by the MIC values 62.5 µg/mL and 125 µg/mL against *C. albicans* and *C. parapsilosis*, respectively.

The effect of replacing the cycloalkyl ring with a carboxylate open chain was investigated in series “**8i**–**iii**”, which revealed an enhanced antifungal activity rather than antibacterial one. The best results were recorded upon incorporating the sulfamethoxazole sulfonamide structure, as in the thienopyrimidine–sulfamethoxazole hybrid **8iii**, which demonstrated mild antibacterial activity only against gram-positive *S. aureus* and gram-negative *E. coli*, recording MICs of 250 µg/mL and 125 µg/mL, respectively. On the other hand, the antifungal activity of the sulfamethoxazole hybrid structure **8iii** was the best among all the tested compounds and considered promising as it demonstrated MICs of 31.25 µg/mL and 62.5 µg/mL against *C. albicans* and *C. parapsilosis*, respectively. These results were better than those of sulfamethoxazole alone (62.5 µg/mL) against both antifungal strains.

Correlating structure to the biological activity upon shifting the substitution from position 4 to position 3, which led to the synthesis of the novel series “**12i**–**iii**”, it was noticed that incorporating sulfadiazine sulfonamide in the thienopyrimidine–sulfadiazine hybrid **12ii** resulted in enhanced antibacterial activity against the bacterial strains *S. aureus* and *E. coli*, which is reflected by its minimum inhibitory concentration (MIC) of 125 µg/mL for both strains. These results were better than those of sulfadiazine alone against gram-positive *S. aureus* (250 µg/mL) but worse than those recorded against gram-negative *E. coli* (31.25 µg/mL). 

Notably, incorporating sulfadiazine into position 4, either in the cyclohexathienopyrimidine core or in its carboxylate analogue in compounds **4ii** and **8ii**, respectively, resulted in an enhanced antifungal activity than with sulfadiazine alone against *Candida albicans* strains and comparable activities against *Candida parapsilosis*, as reflected by their MIC values and inhibition zone values. However, the incorporation of sulfadiazine into position 3 of the cyclohexathieno[2,3-*d*]pyrimidine nucleus in series **12**, as represented in **12ii**, did not improve its antifungal activity. Incorporating sulfamethoxazole into position 4 among all the tested compounds in the three series revealed that compound **8iii** has the highest antifungal activity against *Candida albicans* and *Candida parapsilosis*, with MIC values of 31.25 µg/mL and 62.5 µg/mL, respectively, which are better recorded values than those of both sulfadiazine and sulfamethoxazole when tested alone. It noteworthy to mention that unlike the molecular docking results that reveal the inhibitory activity of theinopyrimidine–sulfaguanidine hybrids to DHFR and SE proteins, they were inactive in all three investigated series even upon changing positions of substitution.

Overall, the results revealed that compounds **8iii**—the thienopyrimidine–sulfamethoxazole hybrid–and **12ii**—the cyclohexathienopyrimidine coupled with sulfadiazine at position 3—showed varying degrees of inhibition against *S. aureus* and *E. coli* bacterial strains, whereas the best antifungal activity against *Candida* strains was displayed by the thienopyrimidine–sulfamethoxazole hybrid **8iii**. Some of the tested compounds showed relatively similar activities close to the references in both growth inhibition zone diameters and MIC values. Although the growth inhibition zone of all active compounds was less than the reference antibiotics, incorporating sulfadiazine into the thienopyrimidine scaffold in compound **12ii** improved its MIC value to higher than that of sulfadiazine itself (Table 3). 

All of the target compounds’ in vitro antifungal efficacies against the examined fungal strains were generally more encouraging than their antibacterial activities. Results of the antibacterial and antifungal activities are shown in Table 3 and Table 4. 

### 2.4. In-Silico Investigation of Physicochemical Properties and Drug Likeness

An in-silico assessment of the synthesized series **4i**–**iii**, **8i**–**iii**, and **12i**–**iii** was performed using both Molsoft and Swiss ADME online web tools [52,53]. Both **4i**–**iii** and **8i**–**iii** were investigated in silico in our previous work [54]. In reference to Lipinski’s rule of five, the number of hydrogen bond acceptors (HBAs) is less than 10, while the number of hydrogen bond donors (HBDs) ranges from two to five in all of the investigated compounds. All the tested compounds recorded iLog *P* < 5, which indicated their high tolerability with cell membranes. In terms of oral bioavailability, although all the screened compounds demonstrated optimum solubility (log *S*) (i.e., not higher than six), lipophilicity did not exceed five, and the number of rotatable bonds was less than or equal to nine, as required. Nonetheless, the compounds are expected to have moderate to poor oral bioavailability. Polarity, in terms of topological polar surface area (TPSA), ranged from 146.38 to 208.68 Å^2^, which suggests moderate to poor oral bioavailability of the compounds, as the optimum TPSA should not exceed 130  Å^2^, as reported [55] (Table 5). 

**Figure 6:**
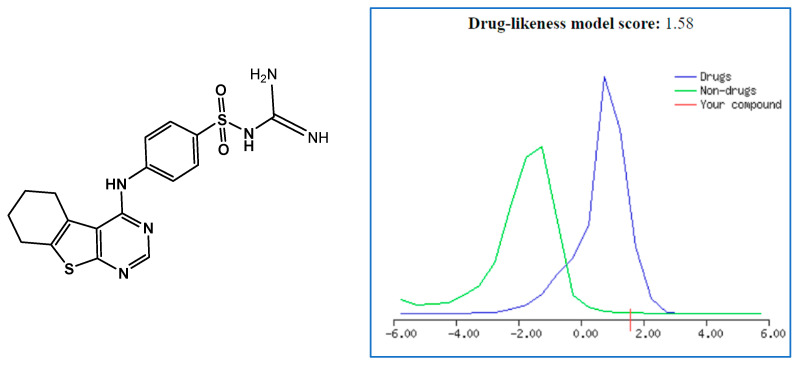
Illustration of the best drug likeness scores of the **4i** compound.

When compounds have positive values, they are deemed promising candidates for drugs, as previously described [45]. The drug likeness scores for the synthesized thienopyrimidine–sulfonamide hybrids ranged from 0.73 to 1.58 (Table 5). Among the hybrids, **4i**, a derivative of the cyclohexathieno[2,3-*d*] pyrimidine core with sulphaguanidine incorporated into position 4, resulted in the best score (1.58), Figure 6. Overall, all the screened compounds are considered promising “drug-like” molecules; none of them violated Lipinski’s rule except for those in series **12**, which demonstrated violations related to the number of electronegative atoms (exceeded 10) and to molecular weight (M.Wt) (slightly exceeded 500) in both **12ii** and **12iii**.

## 3. Materials and Methods

### 3.1. Moelcular Modeling

Molecular Modeling studies were performed using Maestro academic version 2023-4, Molecular Operating Environment (MOE.2022.02) with the aid of Discovery Studio v21.1.0.20298. The crystal structure of the dihydrofolate reductase protein was downloaded from Protein Data Bank (PDB: 2W9S) for the in-silico investigation of antibacterial activity [56], whereas in-silico antifungal screening was performed on the downloaded structure 2AIB targeting squalene peroxidase [57]. Every structure was constructed using MOE Builder, then adjusted, its energy reduced, and saved in mol2 format. The applied protocol for molecular docking was induced fit. The force field for organic molecules was chosen as “MMFF94X”, and the gradient for energy minimization was set to 0.05. Electrostatics, bonding, and Van der Waals forces were all enabled. Calculations were made for partial charges [58].

### 3.2. Chemistry

Using a Stuart SMP10 device, melting points were calculated; the results were not adjusted. The Direct Inlet component of the mass analyzer in the GCMS model with the ISQ single quadrupole thermoscientific Electron Impact mode (UK) was used to perform mass spectra. For the ^1^H NMR spectrum investigation, a Bruker Advance 400 MHz NMR spectrometer was utilized. Tetramethylsilane (TMS) was employed as an internal standard after the chemical shift values were recorded in parts per million. A Bruker Advance 100 MHz spectrometer was used to acquire ^13^C NMR spectra. 

A Gewald reaction was performed to prepare compounds **1** and **5** upon reaction between either cyclohexanone or ethylacetoacetate with sulfur powder, ethylcyanoacetate, and morpholine, to yield **1** and **5**, respectively [59]. Reacting the aminocarboxylate esters **1** and **5** with formamide produced compounds **2** and **6**, respectively, which were then chlorinated via phosphorus oxychloride to yield the chloride derivatives **3** and **7**, respectively [54]. Moreover, compounds **9**–**11** were synthesized according to reported procedures, where a mixture of ethyl 2-(2-amino-4,5,6,7-tetrahydrobenzo[*b*]thiophen-3-yl) acetate (**1**) with acetic anhydride was heated under reflux to yield the thiophene acetamide derivative (**9**). The latter was refluxed with hydrazine hydrate to obtain the thienopyrimidin-4-one derivative (**10**), which resulted in the acetamide derivative (**11**) upon heating with an excess amount of chloroacetyl chloride and drops of triethylamine in dichloromethane [54].

#### 3.2.1. General Procedure for the Synthesis of **4i**–**iii** and **8i**–**iii**

**Scheme 1:**
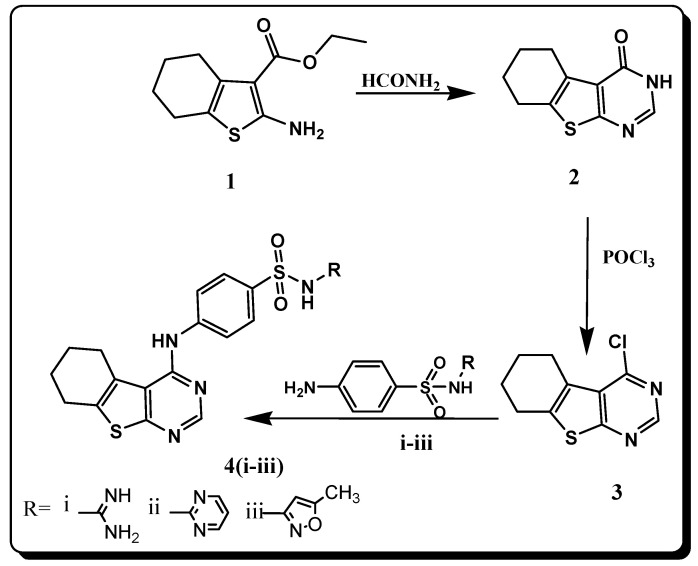
Pathway for synthesis of substituted thienopyrimdine series **4i**–**iii**.

**Scheme 2:**
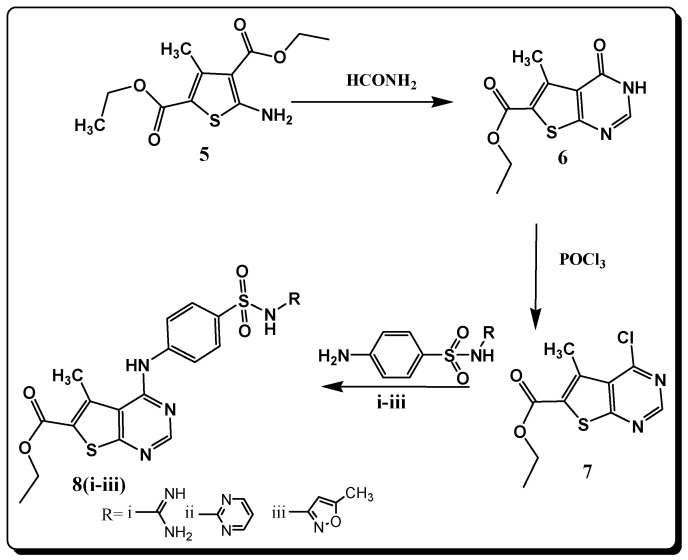
Pathway for synthesis of substituted thienopyrimdine series **8i–iii**.

Equimolar amounts of the chloride derivatives **3** and **7** were refluxed with the appropriate sulfonamides, namely sulphaguanidine, sulfadiazine, and sulfamethoxazole. Reflux was performed in 15 mL of glacial acetic acid for 15 h. The reaction mixture was then left to cool to room temperature before being poured onto ice water. The formed solid was filtered and crystalized from absolute ethanol to yield the **4i**–**iii** and **8**–**i-iii** series, (Scheme 1 and Scheme 2), as reported in our previous investigation [54].

#### 3.2.2. General Procedures for the Synthesis of Series **12i**–**iii**

**Scheme 3:**
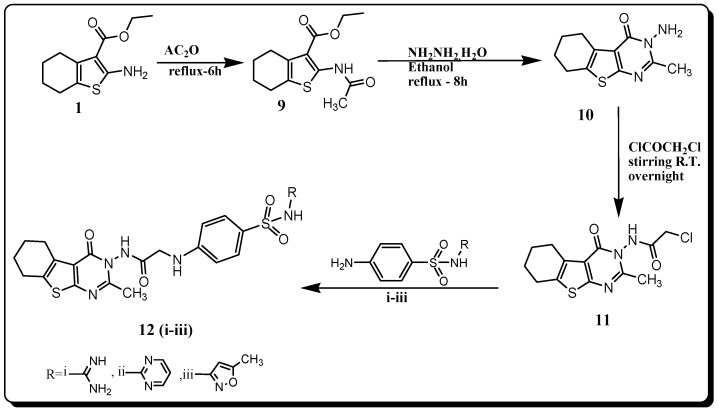
Pathway for synthesis of substituted thienopyrimdine series **12i**–**iii**.

Equimolar amounts of 2-chloro-*N*-(2-methyl-4-oxo-5,6,7,8-tetrahydrobenzo[4,5]thieno[2,3-*d*]pyrimidin-3(4*H*)-yl)acetamide **11** (0.3 g, 0.001 mol) and appropriate sulfonamide derivatives, namely sulphaguanidine, sulfadiazine, and sulfamethoxazole (0.001 mol), were refluxed with stirring in absolute alcohol 15 mL with 3–5 drops of triethylamine (TEA) for 8 h. Then, it was left to cool and poured onto ice/water, crystallized and filtered from glacial acetic acid, and left to dry to obtain compounds **12i**–**iii**, respectively, (Scheme 3). Moreover, all spectroscopic charts for the obtained compounds, **12i–iii**, were also shown in the Supplementary Material.

2-((4-(*N*-carbamimidoylsulfamoyl)phenyl)amino)-*N*-(2-methyl-4-oxo-5,6,7,8-tetrahydrobenzo[4,5]thieno[2,3-*d*] pyrimidin-3(4*H*)-yl) acetamide (**12i**).

m.p. 123–125 °C, yield 67% EI–MS *m*/*z* for: C_22_H_23_N_7_O_4_S_2_ (489.13). ^1^H NMR: δ 1.74, (s, 3H, CH_3_)—1.80–2.81 (m, 8H, cyclohexyl), 3.26 (s, 2H, CH_2_), 4.74 (s, 2H, NH_2_), 5.56 (s, 2H, NH_2_), 6.41 (s, 1H, NH), 7.14–7.57 (m, 4H aromatic), 10.56 (s, 1H, NH), 12.00(s, 1H, NH). ^13^C NMR: δ 21.17, 22.45, 22.58, 25.51, 25.89, 46.47, 112.59, 116.97, 125.21,125.55, 129.88, 131.16,133.91, 139.38, 148.23, 155.85, 157.97, 160.89, 162.66, 168.62. 

*N*-(2-methyl-4-oxo-5,6,7,8-tetrahydrobenzo[4,5]thieno[2,3-*d*]pyrimidin-3(4*H*)-yl)-2-(4-(*N*(pyrimidin-2-yl)sulfamoyl)phenyl)amino)acetamide (**12ii**).

m.p. 150–152 °C, yield 71%. EI–MS *m*/*z* for: C_23_H_23_N_7_O_4_ S_2_ (525.6). ^1^H NMR: δ 1.79 (s, 3H, CH_3_), 1.81–2.84 (m, 8H, cyclohexyl), 3.20 (s, 2H, CH_2_), 4.75 (s, 1H, NH), 5.20 (s, 1H, NH), 7.45–8.29 (m, 4H aromatic ring and 3H- diazine), 11.65 (s, 1H, NH). ^13^C NMR: δ 21.16, 22.45, 22.58, 25.51, 25.89, 46.94, 112.58, 113.52, 116.97,127.38, 129.33, 130.88, 131.30, 133.52, 134.37, 144.78, 148.61, 156.75, 157.86, 157.97, 162.66, 168.62. 

*N*-(2-methyl-4-oxo-5,6,7,8-tetrahydrobenzo[4,5]thieno[2,3-*d*]pyrimidin-3(4*H*)-yl)-2-((4-(*N*-(5-methylisoxazol-3-yl)sulfamoyl)phenyl)amino)acetamide (**12iii**).

m.p. 136–138 °C, yield 62%. EI–MS *m*/*z* for: C_23_H_24_N_6_O_5_S_2_ (528.6). ^1^H NMR: δ 1.07 (s, 3H, CH_3_ of oxazole), 1.79 (s, 3H, CH_3_ of pyrimidine ring), 1.80–2.87 (m, 8H, cyclohexyl), 3.29 (s, 2H, CH_2_), 4.13 (s, 1H-CH oxazole), 4.87 (s, 1H, NH), 5.23 (s, 1H, NH), 7.39–7.94 (m, 4H aromatic), 10.83 (s, 1H, NH). ^13^C NMR: δ 14.09, 22.17, 22.45, 22.58, 25.51, 25.89, 46.47, 95.78, 112.77, 115.90,126.83, 128.90, 129.77, 131.29, 133.56, 138.32, 148.59, 149.44, 157.50, 162.19, 167.50, 169.55. 

### 3.3. Antimicrobial Agents

The antimicrobial agents used were sulfamethoxazole purchased from FUJIFILM Wako Pure Chemical Corporation and sulfadiazine purchased from Titan Biotech. All agents were used as standard antimicrobial agents. Stock solutions (1000 µg/mL) were prepared using 100% dimethyl sulfoxide (DMSO), which was used to dissolve all the reference antimicrobial agents and the tested compounds. 

### 3.4. Organisms

The microbial strains used were provided by King Saud Medical City Central Laboratories. Bacterial strains include gram-positive bacterial strains *Staphylococcus aureus* (ATCC 25923) used for agar-well diffusion, *Staphylococcus aureus* (ATCC 29213) for determining the minimum inhibitory concentration, *Staphylococcus epidermidis* (ATCC 12228), and *Enterococcus faecalis* (ATCC 29212). Gram-negative strains included *Escherichia coli* (ATCC 25922), *Pseudomonas aeruginosa* (ATCC 27853), and *Klebsiella pneumoniae* (ATCC 700603). Fungal strains were additionally tested and included *Candida albicans* (ATCC 10231) and *Candida parapsilosis* (ATCC 22019). All strains were cultured on Mueller Hinton agar and broth and then adjusted to 0.5 McFarland turbidity in 10 mL Mueller Hinton broth for antimicrobial assays. 

### 3.5. Agar Well Diffusion Assay

The antimicrobial activity of the references and new compounds was determined using the agar well diffusion technique [59]. A circle of agar with a diameter of 6 mm was removed from the center of the agar plates to make a well for the addition of the compound solution. The prepared bacterial suspension was inoculated on the surface of the agar plates using a sterile cotton swab. After bacterial inoculation, 100 µL of each antimicrobial and compound (1000 µg/mL) was transferred into the agar well. The plates were incubated aerobically at 37 °C for 18–24 h. The diameters of the inhibition zones were measured around each well and recorded in mm as an average of triplicate experiments. Sulfamethoxazole and sulfadiazine were used as positive controls, and DMSO was used as a negative control. Any compound that showed antimicrobial activity was further tested using the serial dilution susceptibility test for MIC determination.

### 3.6. Serial Dilution Susceptibilty Test

Overnight, bacterial cultures were adjusted to 0.5 McFarland turbidity in 10 mL Mueller Hinton broth, and 150 μL of bacterial suspension was transferred to a 96-well microtiter plate. Two-fold serial dilutions of the applicable antimicrobials and compounds (150 μL) were prepared across the microtiter plate. Negative and positive controls (uninoculated media and a microbial suspension without antimicrobial agents, respectively) were added to the plates and incubated aerobically at 37 °C for 18–24 h. The MIC was described as the lowest concentration of the antimicrobial that prevented the growth of the microorganism. The turbid wells indicated microbial growth, which was compared to the clear negative control. The experiments were carried out in triplicate [60].

### 3.7. In-Silico Investigations

Calculating the drug likeness score of the target compounds was performed using Molsoft, while the investigation of the pharmacokinetics was performed using Swiss ADME online web tools [52,53].

## 4. Conclusions

This work describes the effect of incorporating different sulfonamides into different positions of the thieno[2,3-*d*]pyrimidine scaffold on their antimicrobial activity. To determine the synthesized hybrids’ binding affinity scores to DHFR oxidoreductase and squalene epoxidase proteins, molecular docking studies were carried out, and the outcomes were promising. 

Incorporating different substituted sulfonamide groups into the coplanar structure of thienopyrimidine at position 4 of the cyclohexathienopyrimidine core resulted in mild antibacterial activity. Shifting from position 4 to position 3 demonstrated enhanced antibacterial activity by the thienopyrimidine–sulfadiazine hybrid **12ii** against *S. aureus* bacteria, which was a better result than that of sulfadiazine alone, as reflected by the MIC values. In an attempt to further explore the hybrids’ activity, we investigated the effect of replacing the cycloalkyl ring with a carboxylate open chain, as presented in series **8i**–**iii**, which revealed enhanced antifungal activity compared to the other two series. The best results were recorded by the thienopyrimidine–sulfamethoxazole hybrid **8iii** against both *Candida* strains, and the results were better than those of sulfamethoxazole alone. It is worth mentioning that compared to their antibacterial action, the target compounds’ in vitro antifungal activity against the studied fungal strains was generally more encouraging. Physicochemical properties and drug likeness were assessed in silico, and all the screened compounds were found to be promising drug-like molecules. They all had no Lipinski’s rule violations except those of series **12**, which demonstrated violations related to the number of electronegative atoms (exceeded 10) and to molecular weight (slightly exceeded 500) in both **12ii** and **12iii**.

## Figures and Tables

**Table 1:** Molecular docking results for the thienopyrimidine–sulfonamide hybrids on DHFR reductase (PDB ID: 2W9S).

Compound	Binding Energy (kcal/mol)	RMSD (Å)	Residues Involved in Binding and Types of Interaction
**4i**	−7.1696	1.4085	ASN 18 (H-donor) ASN 18 (H-donor)
**4ii**	−7.8730	1.3052	ASN 18 (H-donor) THR 46 (H-acceptor)
**4iii**	−8.2734	1.7984	GLN 95 (H-acceptor) PHE 92 (H-*pi*)
**8i**	−8.3354	1.2613	ALA 7 (H-acceptor) THR 46 (*pi* H)
**8ii**	−8.3557	1.6653	ASN 18 (H-donor) GLN 95 (H-acceptor) THR 46 (H-acceptor)
**8iii**	−8.1394	1.9062	ASP 27 (A) H-donor
**12i**	−7.2966	1.2439	THR 46 (H-donor) GLN 95(H-acceptor) ASN 18 (*pi* H)
**12ii**	−8.7115	1.3166	THR 46 (H-donor), LYS 45 (H-acceptor), GLN 95 (H-acceptor)
**12iii**	−7.8747	1.6470	THR 46 (H-donor) LYS 45 (*pi*-cation) GLN 95 (*pi* H) THR 96 (*pi* H)

**Table 2:** Molecular docking results for the thienopyrimidin–sulfonamide hybrids on SE protein (PDB ID: 2AIB).

Compound	Binding Energy (kcal/mol)	RMSD (Å)	Residues Involved in Binding and Types of Interaction
**4i**	−7.2835	1.4140	MET 50 (H-donor)
**4ii**	−7.9393	1.2122	THR 74 (H-donor) TYR 47 H-acceptor VAL 75 (*pi*-H) TYR 87 (*pi*-H)
**4iii**	−6.8607	1.3582	THR 74 (H-donor) VAL75 (*pi*-H)
**8i**	−7.6390	1.3130	THR 74 (H-donor)
**8ii**	−7.9635	1.4180	MET 35 (*pi* H)
**8iii**	−8.2032	1.8383	TYR 47 (H-donor) TYR 47 (H-donor)
**12i**	−8.3712	1.8964	TYR 12 (H-donor) LEU 19 (*pi* H)
**12ii**	−9.3391	1.7258	MET 50 (H-donor) TYR 47 (H-acceptor)
**12iii**	−9.1466	1.4663	MET 50 (H-donor) TYR 12 (H-donor)

**Table 3:** Antibacterial activity of the synthesized theinopyrimidine–sulfonamides hybrids.

Compounds	Gram-Positive Bacteria						Gram-Negative Bacteria					
	*S. aureus*		*S. epidermidis*		*E. faecalis*		*E. coli*		*P. aeruginosa*		*K. pneumoniae*	
	*GIZ	MIC	*GIZ	MIC	*GIZ	MIC	*GIZ	MIC	*GIZ	MIC	*GIZ	MIC
**4i**	0	-	0	-	0	-	0		0	-	0	-
**4ii**	15.33 ± 1.15	500	0	-	0	-	18 ± 0	125	0	-	0	-
**4iii**	0	-	0	-	0	-	0	-	0	-	0	-
**8i**	0	-	0	-	0	-	0	-	0	-	0	-
**8ii**	16 ± 1	500	0	-	0	-	17 ± 0	125	0	-	0	-
**8iii**	22.33 ± 0.58	250	0	-	0	-	17.33 ± 0.58	125	0	-	0	-
**12i**	0	-	0	-	0	-	0		0	-	0	-
**12ii**	24.67 ± 0.58	125	0	-	0	-	23.67 ± 1.53	125	0	-	0	-
**12iii**	19 ± 1	250	0	-	0	-	19.67 ± 0.58	125	0	-	0	-
**Sulfadiazine**	29.67 ± 0.58	250	0	-	0	-	27.67 ± 1.15	31.25	0	-	0	-
**Sulfamethoxazole**	37.67 ± 1.15	125	15 ± 0	-	0	-	30.67 ± 1.15	15.625	0	-	0	-

*GIZ: average diameter (mm) of growth inhibition zone in mean ± SD; MIC: minimum inhibitory concentration (µg/mL); number of replicates *n* = 3.

**Table 4:** Antifungal activity of the synthesized compounds against *Candida* strains.

Compounds	*C. albicans*		*C. parapsilosis*	
	*GIZ	MIC	*GIZ	MIC
**4i**	0	-	0	-
**4ii**	9 ± 0	62.5	8.33 ± 0.58	125
**4iii**	0	-	0	-
**8i**	0	-	0	-
**8ii**	9.33 ± 0.58	62.5	9 ± 0	125
**8iii**	17.67 ± 0.58	31.25	15.67 ± 0.58	62.5
**12i**	11.67 ± 1.15	125	10.33 ± 0.58	125
**12ii**	7.67 ± 0.58	250	8.67 ± 0.58	500
**12iii**	10.33 ± 0.58	125	9.33 ± 0.58	125
**Sulfadiazine**	8.67 ± 1.15	125	9 ± 0	125
**Sulfamethoxazole**	10.67 ± 0.58	62.5	10.67 ± 0.58	62.5

*GIZ: average diameter (mm) of growth inhibition zone in mean ± SD; MIC: minimum inhibitory concentration (µg/mL); number of replicates *n* = 3.

**Table 5:** Predicted physicochemical properties and drug-like candidates.

Compounds	M.Wt	HBA	HBD	TPSA	Rotatable Bonds	Lipophilicity	Log S	iLog *P*	Lipinski Violations	Drug Likeness
**4i**	402.49	5	4	170.47	5	2.92	−4.26	1.51	0	1.58
**4ii**	438.53	6	2	146.38	5	3.8	−5.14	2.66	0	1.03
**4iii**	441.53	6	2	146.63	5	4.36	−5.49	2.59	0	1.08
**8i**	434.49	7	4	196.77	8	2.61	−4.03	1.75	0	1.19
**8ii**	470.52	8	2	172.68	8	3.48	−4.91	2.78	0	0.88
**8iii**	473.53	8	2	172.93	8	4.05	−5.26	3.1	0	0.73
**12i**	489.13	6	5	208.68	8	1.6	−3.69	2	1	1.23
**12ii**	525.60	7	3	184.59	8	2.47	−4.56	2.23	2	1.35
**12iii**	528.60	7	3	184.84	8	3.04	−4.92	2.11	2	1.06

## Data Availability

The authors confirm that all the data supporting this study are available within the article.

## Supplementary Materials

The following supporting information can be downloaded at: https://www.mdpi.com/article/10.3390/ph17020188/s1, spectroscopic charts for **12i**–**iii**.

## References

[1] Ahmed M., Sayed M., Saber A.F., Hassanien R., Kamal El-Dean A.M., Tolba M.S. (2022). Synthesis, characterization, and antimicrobial activity of new thienopyrimidine derivatives. Polycycl. Aromat. Compd..

[2] Rashad A.E., Shamroukh A.H., Abdel-Megeid R.E., Mostafa A., El-Shesheny R., Kandeil A., Ali M.A., Banert K. (2010). Synthesis and screening of some novel fused thiophene and thienopyrimidine derivatives for anti-avian influenza virus (H5N1) activity. Eur. J. Med. Chem..

[3] Tolba M.S., Sayed A.M., Sayed M., Ahmed M. (2021). Design, synthesis, biological evaluation, and molecular docking of some new Thieno[2,3-*d*] pyrimidine derivatives. J. Mol. Struct..

[4] Tolba M.S., Ahmed M., Kamal El-Dean A.M., Hassanien R., Farouk M. (2018). Synthesis of New Fused Thienopyrimidines Derivatives as Anti-Inflammatory Agents. J. Heterocycl. Chem..

[5] Tasler S., Baumgartner R., Ammendola A., Schachtner J., Wieber T., Blisse M., Rath S., Zaja M., Klahn P., Quotschalla U. (2010). Thienopyrimidines as β3-adrenoceptor agonists: Hit-to-lead optimization. Bioorg. Med. Chem. Lett..

[6] Li S., Vilchèze C., Chakraborty S., Wang X., Kim H., Anisetti M., Ekins S., Rhee K.Y., Jacobs W.R., Freundlich J.S. (2015). Evolution of a thienopyrimidine antitubercular relying on medicinal chemistry and metabolomics insights. Tetrahedron Lett..

[7] Bell A.S., Yu Z., Hutton J.A., Wright M.H., Brannigan J.A., Paape D., Roberts S.M., Sutherell C.L., Ritzefeld M., Wilkinson A.J. (2020). Novel thienopyrimidine inhibitors of Leishmania N-myristoyltransferase with on-target activity in intracellular amastigotes. J. Med. Chem..

[8] Ghith A., Ismail N.S.M., Youssef K., Abouzid K.A.M. (2017). Medicinal Attributes of Thienopyrimidine Based Scaffold Targeting Tyrosine Kinases and Their Potential Anticancer Activities. Arch. Pharm..

[9] Elmongy E.I. (2020). Thieno[2,3-*d*] pyrimidine derivatives: Synthetic approaches and their FLT3 kinase inhibition. J. Heterocycl. Chem..

[10] Elmongy E.I., Altwaijry N., Attallah N.G., AlKahtani M.M., Henidi H.A. (2022). In-silico screening of novel synthesized thienopyrimidines targeting fms related receptor tyrosine kinase-3 and their in-vitro biological evaluation. Pharmaceuticals.

[11] Elmongy E.I., Henidi H.A. (2022). In Silico Evaluation of a Promising Key Intermediate Thieno[2,3-d] Pyrimidine Derivative with Expected JAK2 Kinase Inhibitory Activity. Molbank.

[12] Elsebaie H.A., El-Moselhy T.F., El-Bastawissy E.A., Elberembally K.M., Badi R.M., Elkaeed E.B., Shaldam M.A., Eldehna W.M., Tawfik H.O. (2024). Development of new thieno[2,3-*d*]pyrimidines as dual EGFR and STAT3 inhibitors endowed with anticancer and pro-apoptotic activities. Bioorg. Chem..

[13] Elsayed S., Abdelkhalek A.S., Rezq S., Abu Kull M.E., Romero D.G., Kothayer H. (2023). Magic shotgun approach to anti-inflammatory pharmacotherapy: Synthesis of novel thienopyrimidine monomers/heterodimer as dual COX-2 and 15-LOX inhibitors endowed with potent antioxidant activity. Eur. J. Med. Chem..

[14] Elmongy E., Kedr M., Abotaleb N., Abbas S. (2021). Design and synthesis of new thienopyrimidine derivatives along with their antioxidant activity. Egypt. J. Chem..

[15] Eissa K.I., Kamel M.M., Mohamed L.W., Doghish A.S., Alnajjar R., Al-Karmalawy A.A., Kassab A.E. (2023). Design, synthesis, and biological evaluation of thienopyrimidine derivatives as multifunctional agents against Alzheimer’s disease. Drug Dev. Res..

[16] Kotaiah Y., Harikrishna N., Nagaraju K., Rao C.V. (2012). Synthesis and antioxidant activity of 1,3,4-oxadiazole tagged thieno [2,3-*d*] pyrimidine derivatives. Eur. J. Med. Chem..

[17] Sharaky M., Kamel M., Aziz M.A., Omran M., Rageh M.M., Abouzid K.A., Shouman S.A. (2020). Design, synthesis and biological evaluation of a new thieno[2,3-*d*]pyrimidine-based urea derivative with potential antitumor activity against tamoxifen sensitive and resistant breast cancer cell lines. J. Enzym. Inhib. Med. Chem..

[18] Elmongy E.I., Attallah N.G.M., Altwaijry N., AlKahtani M.M., Henidi H.A. (2021). Design and synthesis of new thiophene/thieno[2,3-d]pyrimidines along with their cytotoxic biological evaluation as tyrosine kinase inhibitors in addition to their apoptotic and autophagic induction. Molecules.

[19] Sayed M.T.M., Hassan R.A., Halim P.A., El-Ansary A.K. (2023). Recent updates on thienopyrimidine derivatives as anticancer agents. Med. Chem. Res..

[20] Abu-Hashem A.A., Abu-Zied K.M., AbdelSalam Zaki M.E., El-Shehry M.F., Awad H.M., Khedr M.A. (2020). Design, synthesis, and anticancer potential of the enzyme (PARP-1) inhibitor with computational studies of new triazole, thiazolidinone, -thieno [2,3-d] pyrimidinones. Lett. Drug Des. Discov..

[21] Kousovista R., Athanasiou C., Liaskonis K., Ivopoulou O., Karalis V. (2021). Association of antibiotic use with the resistance epidemiology of *Pseudomonas aeruginosa* in a hospital setting: A four-year retrospective time series analysis. Sci. Pharm..

[22] Sirakanyan S.N., Kartsev V.G., Geronikaki A., Spinelli D., Petrou A., Hakobyan E.K., Glamoclija J., Ivanov M., Sokovic M., Hovakimyan A.A. (2020). Synthesis and Evaluation of Antimicrobial Activity and Molecular Docking of New N-1, 3-thiazol-2-ylacetamides of Condensed Pyrido [3′,2′:4,5] furo (thieno)[3,2-d] pyrimidines. Curr. Top. Med. Chem..

[23] Sirakanyan S.N., Geronikaki A., Spinelli D., Hakobyan E.K., Kartsev V.G., Petrou A., Hovakimyan A.A. (2018). Synthesis and antimicrobial activity of new amino derivatives of pyrano [4″,3″:4′,5′] pyrido [3′,2′:4,5] thieno [3,2-d] pyrimidine. An. Acad. Bras. Ciências.

[24] Aruna Kumari M., Triloknadh S., Harikrishna N., Vijjulatha M., Venkata Rao C. (2017). Synthesis, Antibacterial Activity, and Docking Studies of 1,2,3-triazole-tagged Thieno [2,3-*d*] pyrimidinone Derivatives. J. Heterocycl. Chem..

[25] Lagardère P., Fersing C., Masurier N., Lisowski V. (2021). Thienopyrimidine: A promising Scaffold to access anti-infective agents. Pharmaceuticals.

[26] Ezabadi I.R., Camoutsis C., Zoumpoulakis P., Geronikaki A., Soković M., Glamočilija J., Ćirić A. (2008). Sulfonamide-1,2,4-triazole derivatives as antifungal and antibacterial agents: Synthesis, biological evaluation, lipophilicity, and conformational studies. Bioorg. Med. Chem..

[27] Mohi El-Deen E.M., Anwar M.M., El-Gwaad A.A.A., Karam E.A., El-Ashrey M.K., Kassab R.R. (2022). Novel pyridothienopyrimidine derivatives: Design, synthesis and biological evaluation as antimicrobial and anticancer agents. Molecules.

[28] Ibrahim H.S., Eldehna W.M., Abdel-Aziz H.A., Elaasser M.M., Abdel-Aziz M.M. (2014). Improvement of antibacterial activity of some sulfa drugs through linkage to certain phthalazin-1(2*H*)-one scaffolds. Eur. J. Med. Chem..

[29] Capasso C., Supuran C.T. (2014). Sulfa and trimethoprim-like drugs–antimetabolites acting as carbonic anhydrase, dihydropteroate synthase and dihydrofolate reductase inhibitors. J. Enzym. Inhib. Med. Chem..

[30] Chibale K., Haupt H., Kendrick H., Yardley V., Saravanamuthu A., Fairlamb A.H., Croft S.L. (2001). Antiprotozoal and cytotoxicity evaluation of sulfonamide and urea analogues of quinacrine. Bioorg. Med. Chem. Lett..

[31] Scarim C.B., Chelucci R.C., Dos Santos J.L., Chin C.M. (2020). The use of Sulfonamide Derivatives in the Treatment of Trypanosomatid Parasites including Trypanosoma cruzi, Trypanosoma brucei, and Leishmania ssp. Med. Chem..

[32] Xu Z.-Q., Flavin M.T., Flavin J. (2014). Combating multidrug-resistant Gram-negative bacterial infections. Expert Opin. Investig. Drugs.

[33] Zakšauskas A., Čapkauskaitė E., Jezepčikas L., Linkuvienė V., Paketurytė V., Smirnov A., Leitans J., Kazaks A., Dvinskis E., Manakova E. (2020). Halogenated and di-substituted benzenesulfonamides as selective inhibitors of carbonic anhydrase isoforms. Eur. J. Med. Chem..

[34] Ammar Y.A., El-Sharief A.M.S., Belal A., Abbas S.Y., Mohamed Y.A., Mehany A.B., Ragab A. (2018). Design, synthesis, antiproliferative activity, molecular docking and cell cycle analysis of some novel (morpholinosulfonyl) isatins with potential EGFR inhibitory activity. Eur. J. Med. Chem..

[35] El-Sharief A.M.S., Ammar Y.A., Belal A., El-Sharief M.A.S., Mohamed Y.A., Mehany A.B., Ali G.A.E., Ragab A. (2019). Design, synthesis, molecular docking and biological activity evaluation of some novel indole derivatives as potent anticancer active agents and apoptosis inducers. Bioorg. Chem..

[36] Isik S., Kockar F., Aydin M., Arslan O., Guler O.O., Innocenti A., Scozzafava A., Supuran C.T. (2009). Carbonic anhydrase inhibitors: Inhibition of the β-class enzyme from the yeast Saccharomyces cerevisiae with sulfonamides and sulfamates. Bioorg. Med. Chem..

[37] Bouissane L., El Kazzouli S., Léonce S., Pfeiffer B., Rakib E., Khouili M., Guillaumet G. (2006). Synthesis and biological evaluation of N-(7-indazolyl) benzenesulfonamide derivatives as potent cell cycle inhibitors. Bioorg. Med. Chem..

[38] Camoutsis C., Geronikaki A., Ciric A., Soković M., Zoumpoulakis P., Zervou M. (2010). Sulfonamide-1,2,4-thiadiazole derivatives as antifungal and antibacterial agents: Synthesis, biological evaluation, lipophilicity, and conformational studies. Chem. Pharm. Bull..

[39] Weber A., Casini A., Heine A., Kuhn D., Supuran C.T., Scozzafava A., Klebe G. (2004). Unexpected nanomolar inhibition of carbonic anhydrase by COX-2-selective celecoxib: New pharmacological opportunities due to related binding site recognition. J. Med. Chem..

[40] Ammar Y.A., El-Sharief A., Mohamed Y.A., Mehany A.B., Ragab A. (2018). Synthesis, spectral characterization and pharmacological evaluation of novel thiazole-oxoindole hybrid compounds as potential anticancer agents. Al-Azhar Bull. Sci..

[41] Penning T.D., Talley J.J., Bertenshaw S.R., Carter J.S., Collins P.W., Docter S., Graneto M.J., Lee L.F., Malecha J.W., Miyashiro J.M. (1997). Synthesis and biological evaluation of the 1, 5-diarylpyrazole class of cyclooxygenase-2 inhibitors: Identification of 4-[5-(4-methylphenyl)-3-(trifluoromethyl)-1H-pyrazol-1-yl]benzenesulfonamide (SC-58635, celecoxib). J. Med. Chem..

[42] Castaño L.F., Quiroga J., Abonia R., Insuasty D., Vidal O.M., Seña R., Rubio V., Puerto G., Nogueras M., Cobo J. (2022). Synthesis, Anticancer and Antitubercular Properties of New Chalcones and Their Nitrogen-Containing Five-Membered Heterocyclic Hybrids Bearing Sulfonamide Moiety. Int. J. Mol. Sci..

[43] Eldeeb M., Sanad E.F., Ragab A., Ammar Y.A., Mahmoud K., Ali M.M., Hamdy N.M. (2022). Anticancer effects with molecular docking confirmation of newly synthesized isatin sulfonamide molecular hybrid derivatives against hepatic cancer cell lines. Biomedicines.

[44] Ragab A., Fouad S.A., Ali O.A.A., Ahmed E.M., Ali A.M., Askar A.A., Ammar Y.A. (2021). Sulfaguanidine hybrid with some new pyridine-2-one derivatives: Design, synthesis, and antimicrobial activity against multidrug-resistant bacteria as dual DNA gyrase and DHFR inhibitors. Antibiotics.

[45] Zaidi S.L., Agarwal S.M., Chavalitshewinkoon-Petmitr P., Suksangpleng T., Ahmad K., Avecilla F., Azam A. (2016). Thienopyrimidine sulphonamide hybrids: Design, synthesis, antiprotozoal activity and molecular docking studies. RSC Adv..

[46] Sławiński J., Żołnowska B., Pirska D., Kędzia A., Kwapisz E. (2013). Synthesis and antibacterial activity of novel 4-chloro-2-mercaptobenzenesulfonamide derivatives. J. Enzym. Inhib. Med. Chem..

[47] Vlasov S.V., Vlasova O.D., Severina H.I., Krolenko K.Y., Borysov O.V., Abu Sharkh A.I.M., Vlasov V.S., Georgiyants V.A. (2021). Design, Synthesis and In Vitro Antimicrobial Activity of 6-(1*H*-Benzimidazol-2-yl)-3,5-dimethyl-4-oxo-2-thio-3,4-dihydrothieno [2,3-*d*]pyrimidines. Sci. Pharm..

[48] Ovung A., Bhattacharyya J. (2021). Sulfonamide drugs: Structure, antibacterial property, toxicity, and biophysical interactions. Biophys. Rev..

[49] Teng X., Wang Y., Gu J., Shi P., Shen Z., Ye L. (2018). Antifungal agents: Design, synthesis, antifungal activity and molecular docking of phloroglucinol derivatives. Molecules.

[50] Malwal S.R., Shang N., Liu W., Li X., Zhang L., Chen C.-C., Guo R.-T., Oldfield E. (2022). A Structural and Bioinformatics Investigation of a Fungal Squalene Synthase and Comparisons with Other Membrane Proteins. ACS Omega.

[51] Prabhakar V., Babu K.S., Ravindranath L., Venkateswarlu B. (2017). Synthesis and biological activities of novel thieno[3,2-*d*] pyrimidine derivatives. Asian J. Res. Chem..

[52] Daina A., Michielin O., Zoete V. (2017). SwissADME: A free web tool to evaluate pharmacokinetics, drug-likeness and medicinal chemistry friendliness of small molecules. Sci. Rep..

[53] Fields U.A.P. (2012). MolSoft ICM Quarterly.

[54] Elmongy E.I., Binjubair F.A., Alshehri O.Y., Baeshen K.A., Almukhalfi Z.A., Henidi H.A. (2023). In Silico Screening and Anticancer-Apoptotic Evaluation of Newly Synthesized Thienopyrimidine/Sulfonamide Hybrids. Int. J. Mol. Sci..

[55] Ritchie T.J., Ertl P., Lewis R. (2011). The graphical representation of ADME-related molecule properties for medicinal chemists. Drug Discov. Today.

[56] Heaslet H., Harris M., Fahnoe K., Sarver R., Putz H., Chang J., Subramanyam C., Barreiro G., Miller J.R. (2009). Structural comparison of chromosomal and exogenous dihydrofolate reductase from Staphylococcus aureus in complex with the potent inhibitor trimethoprim. Proteins Struct. Funct. Bioinform..

[57] Rodrigues M.L., Archer M., Martel P., Miranda S., Thomaz M., Enguita F.J., Baptista R.P., Pinho e Melo E., Sousa N., Cravador A. (2006). Crystal structures of the free and sterol-bound forms of β-cinnamomin. Biochim. Biophys. Acta (BBA)—Proteins Proteom..

[58] Elmongy E.I., Ahmed A.A.S., El Sayed I.E.T., Fathy G., Awad H.M., Salman A.U., Hamed M.A. (2022). Synthesis, Biocidal and Antibiofilm Activities of New Isatin–Quinoline Conjugates against Multidrug-Resistant Bacterial Pathogens along with Their In-Silico Screening. Antibiotics.

[59] Gewald K., Schinke E., Böttcher H. (1966). Heterocyclen aus CH-aciden Nitrilen, VIII. 2-Amino-thiophene aus methylenaktiven Nitrilen, Carbonylverbindungen und Schwefel. Chem. Berichte.

[60] Al-Wabli R.I., Alsulami M.A., Bukhari S.I., Moubayed N.M.S., Al-Mutairi M.S., Attia M.I. (2021). Design, Synthesis, and Antimicrobial Activity of Certain New Indole-1,2,4 Triazole Conjugates. Molecules.

