# Supplementary material for: Antimicrobial Evaluation of Sulfonamides after Coupling with Thienopyrimidine Coplanar Structure
Source: Pharmaceuticals (Basel). 2024 Jan 31;17(2):188. doi: 10.3390/ph17020188 (PMC10892651; doi:10.3390/ph17020188)
Supplement: Supplementary file 1 [file pharmaceuticals-17-00188-s001.zip › pharmaceuticals-2814440-supplementary.pdf]

Antimicrobial evaluation of sulfonamides after coupling with thienopyrimidines coplanar structure

Elshaymaa I. Elmongy [1], Wejdan S. Alanazi [2], Alhanouf I. Aldawsari [2], Asma A. Alfaouri[2], Reem Binsuwaidan[3*]

1 Department of Pharmaceutical Chemistry, Faculty of Pharmacy, Helwan University, Ain Helwan, Cairo P.O. Box 11795, Egypt; shaymaa.taha@pharm.helwan.edu.eg
2 College of Pharmacy, Princess Nourah bint Abdulrahman University, P.O. Box 84428, Riyadh 11671, Saudi Arabia; wejdan.saad18@gmail.com; hanouff568@gmail.com; asma.ayed.al@gmail.com
3 Department of Pharmaceutical Sciences, College of Pharmacy, Princess Nourah bint Abdulrahman University, P.O. Box 84428, Riyadh 11671, Saudi Arabia; rabinsuwaidan@pnu.edu.sa
* Correspondence: rabinsuwaidan@pnu.edu.sa

1H NMR

12i-

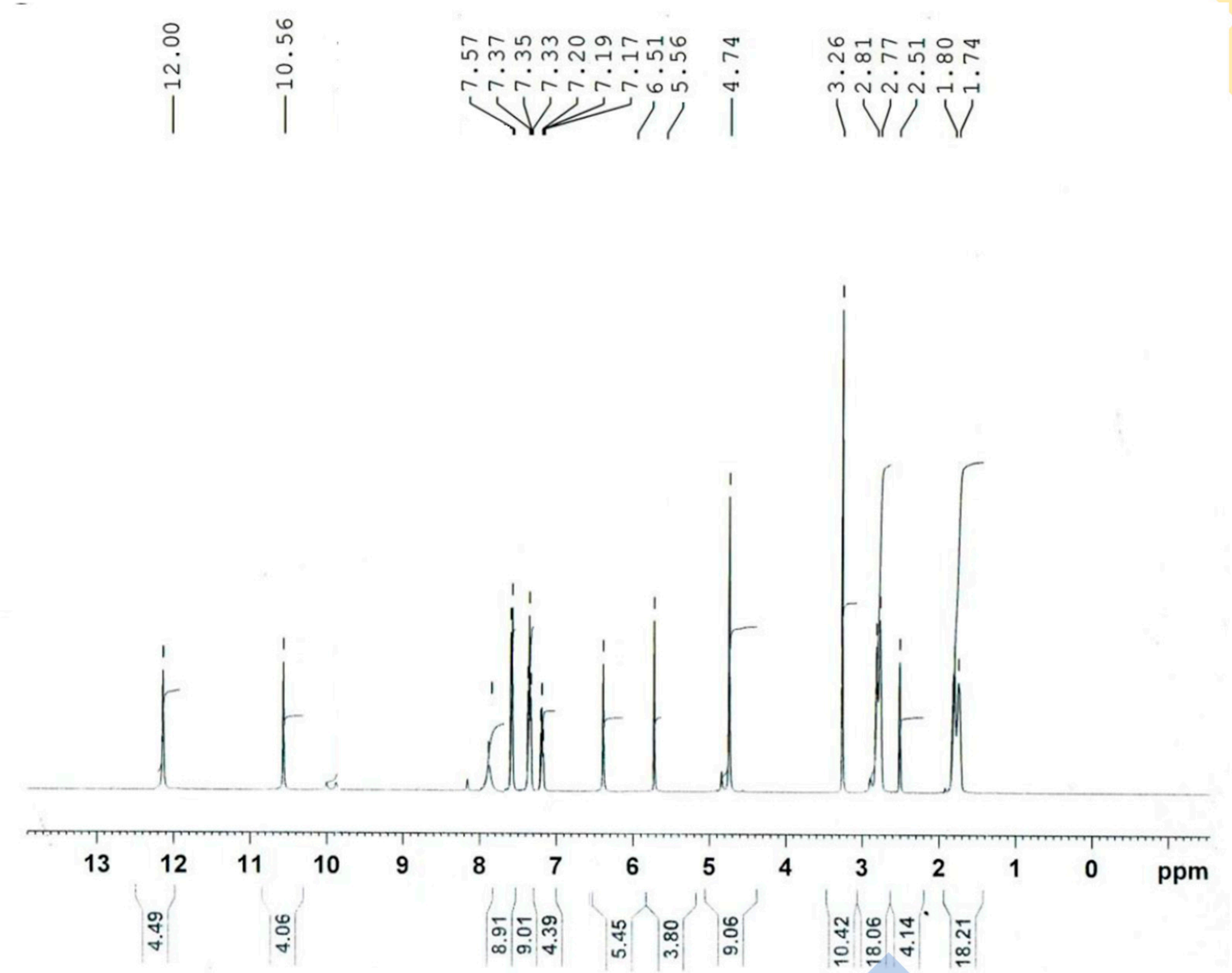

12.00
10.56
7.57
7.37
7.35
7.33
7.20
7.19
7.17
6.51
5.56
4.74
3.26
2.81
2.77
2.51
1.80
1.74
13
12
11
10
9
8
7
6
5
4
3
2
1
0
ppm
4.49
4.06
8.91
9.01
4.39
5.45
3.80
9.06
10.42
18.06
4.14
18.21

12II

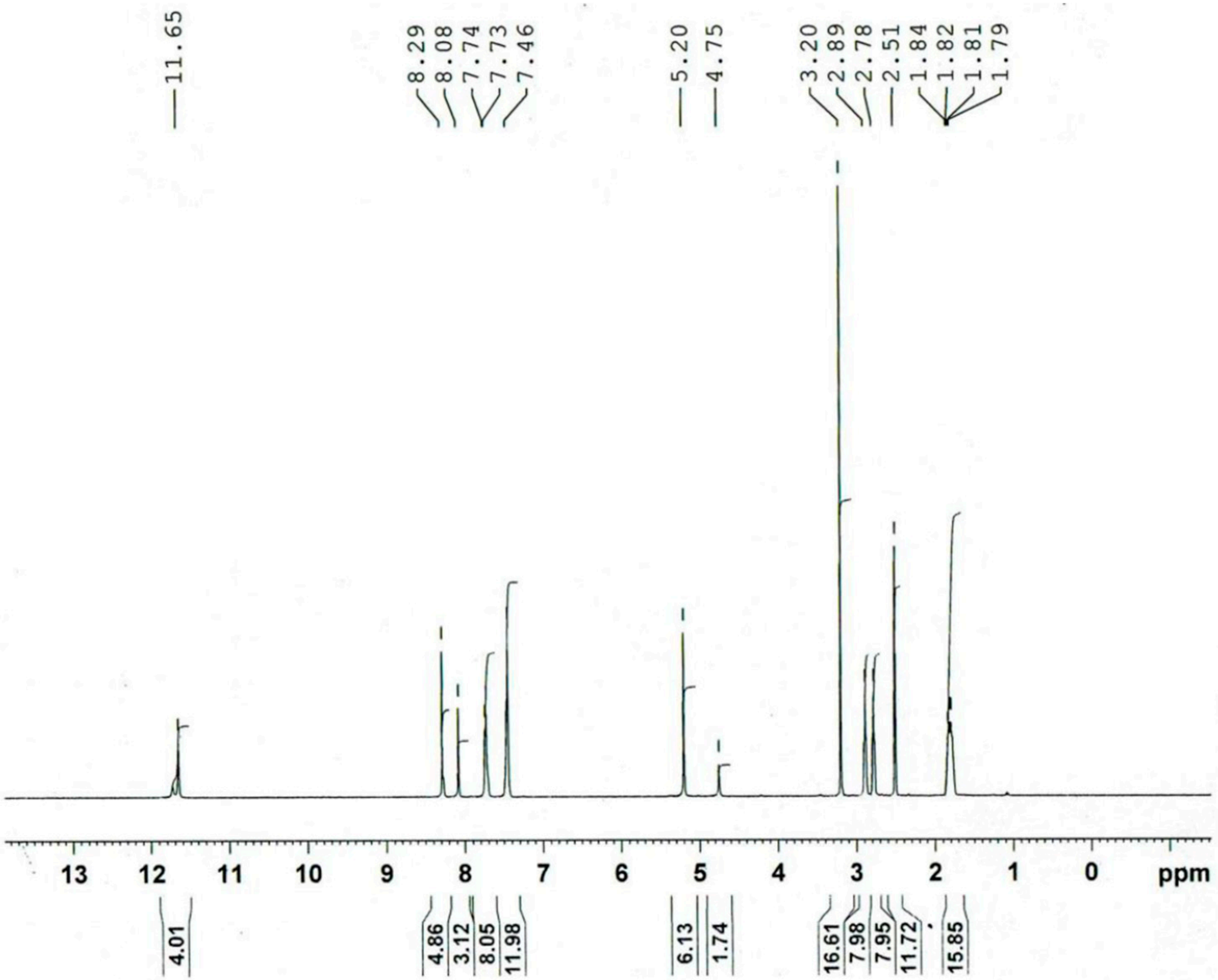
11.65
8.29
8.08
7.74
7.73
7.46
5.20
4.75
3.20
2.89
2.78
2.51
1.84
1.82
1.81
1.79
13 12 11 10 9 8 7 6 5 4 3 2 1 0 ppm
4.01
4.86
3.12
8.05
11.98
6.13
1.74
16.61
7.98
7.95
11.72
15.85

12III

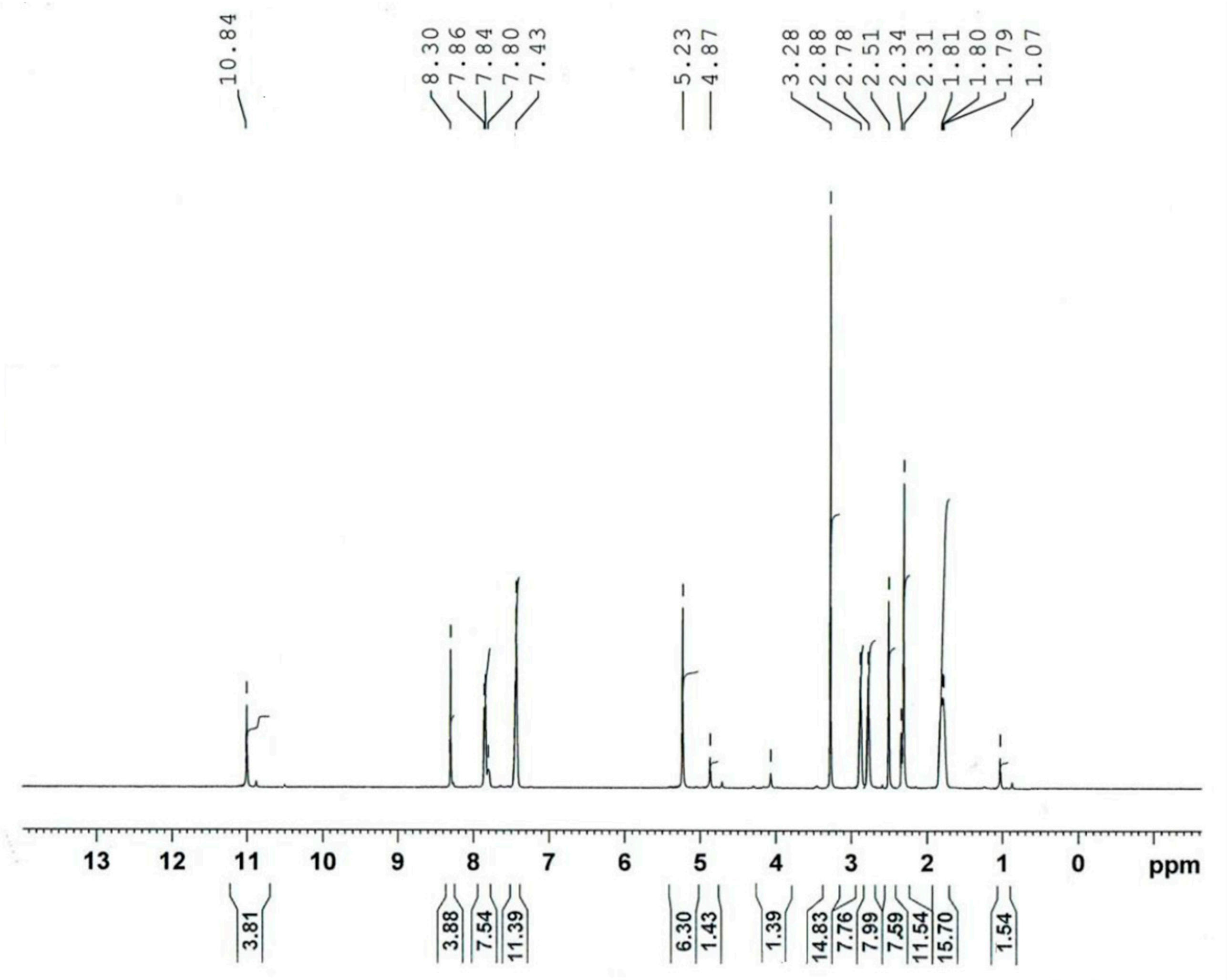
10.84
8.30
7.86
7.84
7.80
7.43
5.23
4.87
3.28
2.88
2.78
2.51
2.34
2.31
1.81
1.80
1.79
1.07
13 12 11 10 9 8 7 6 5 4 3 2 1 0 ppm
3.81
3.88
7.54
11.39
6.30
1.43
1.39
14.83
7.76
7.99
7.59
11.54
15.70
1.54

C-13 NMR

12l

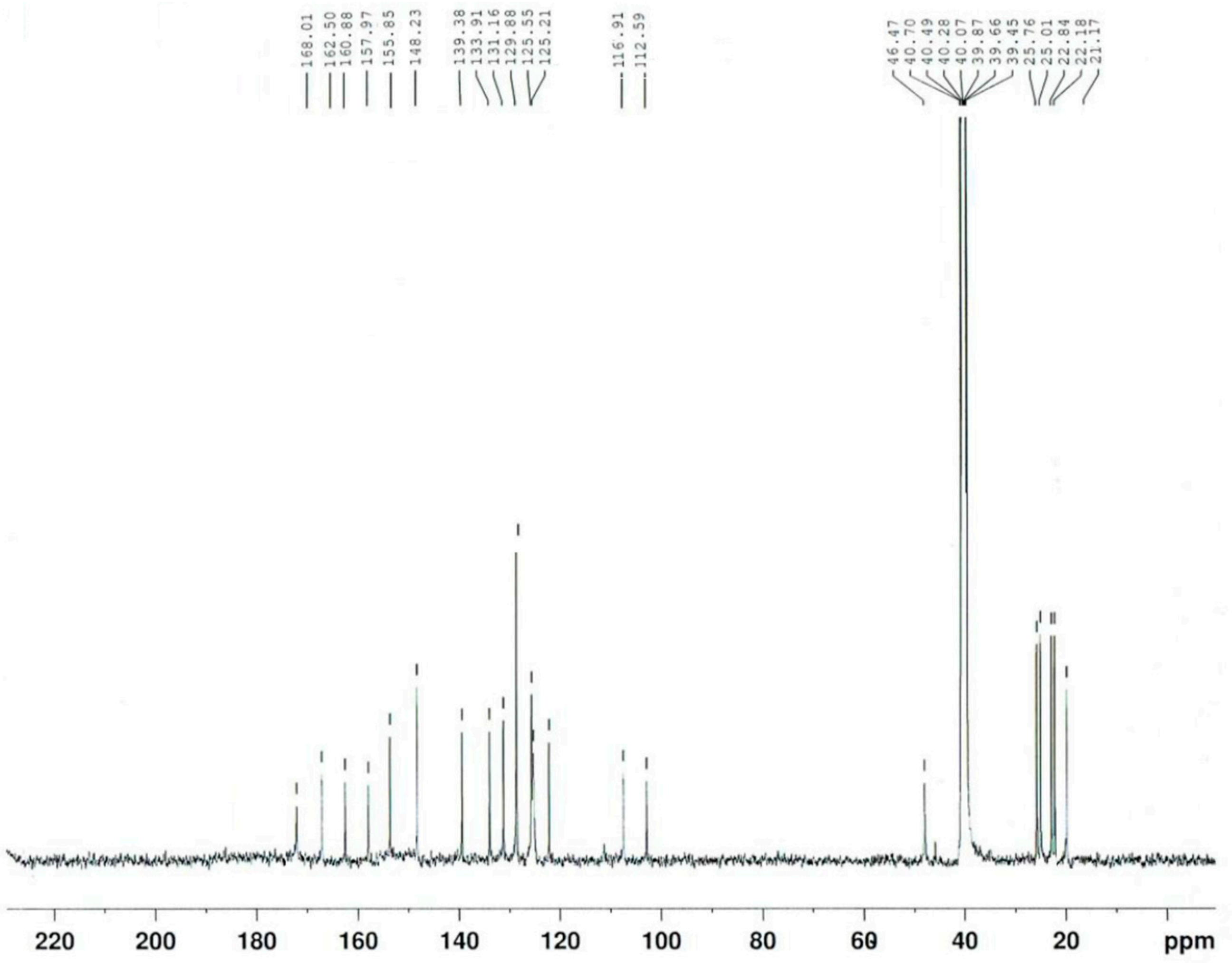
168.01
162.50
160.88
157.97
155.85
148.23
139.38
133.91
131.16
129.88
125.55
125.21
116.91
112.59
46.47
40.70
40.49
40.28
40.07
39.87
39.66
39.45
25.76
25.01
22.84
22.18
21.17
220
200
180
160
140
120
100
80
60
40
20
ppm

12II

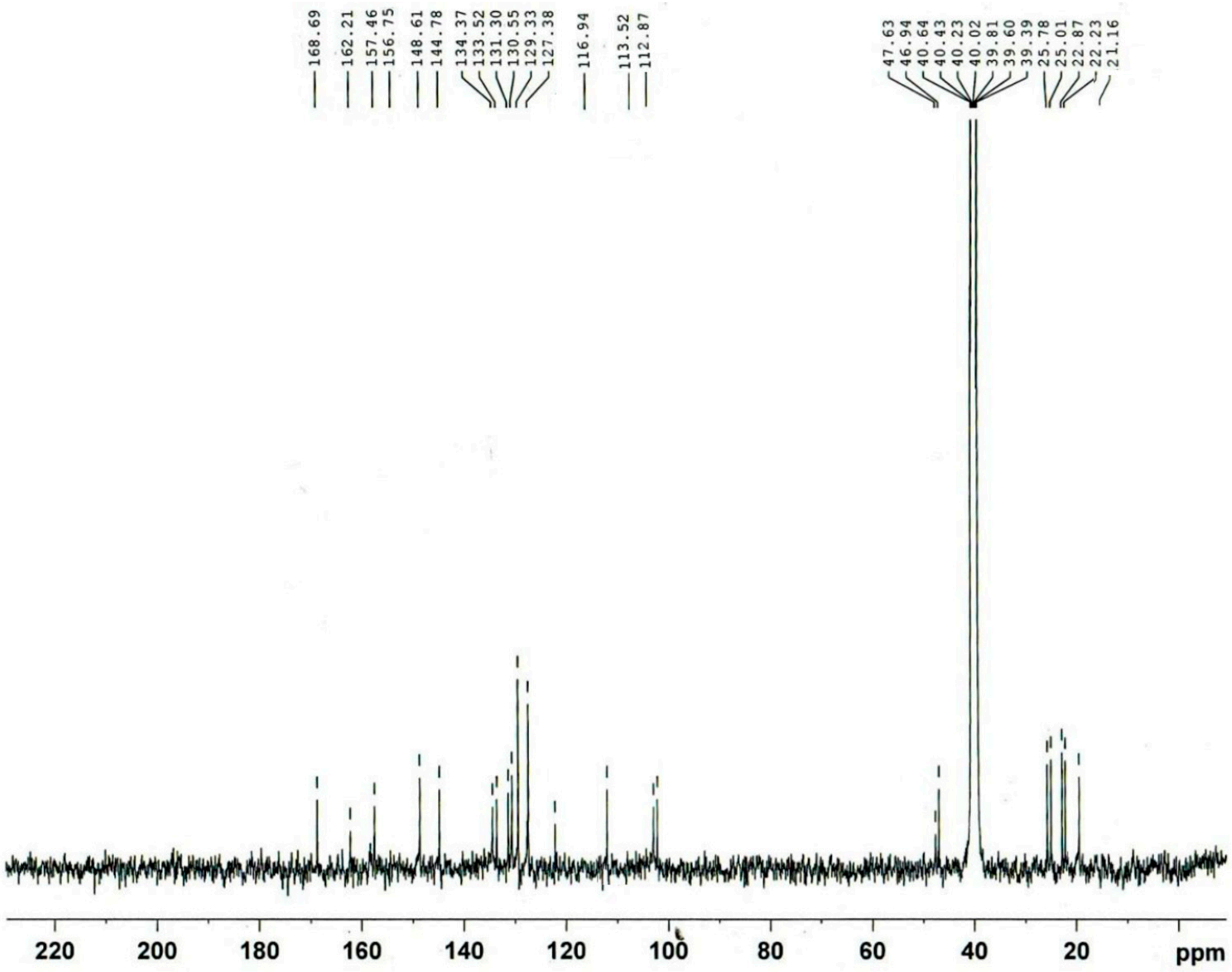
168.69
162.21
157.46
156.75
148.61
144.78
134.37
133.52
131.30
130.55
129.33
127.38
116.94
113.52
112.87
47.63
46.94
40.64
40.43
40.23
40.02
39.81
39.60
39.39
25.78
25.01
22.87
22.23
21.16
220
200
180
160
140
120
100
80
60
40
20
ppm

12III

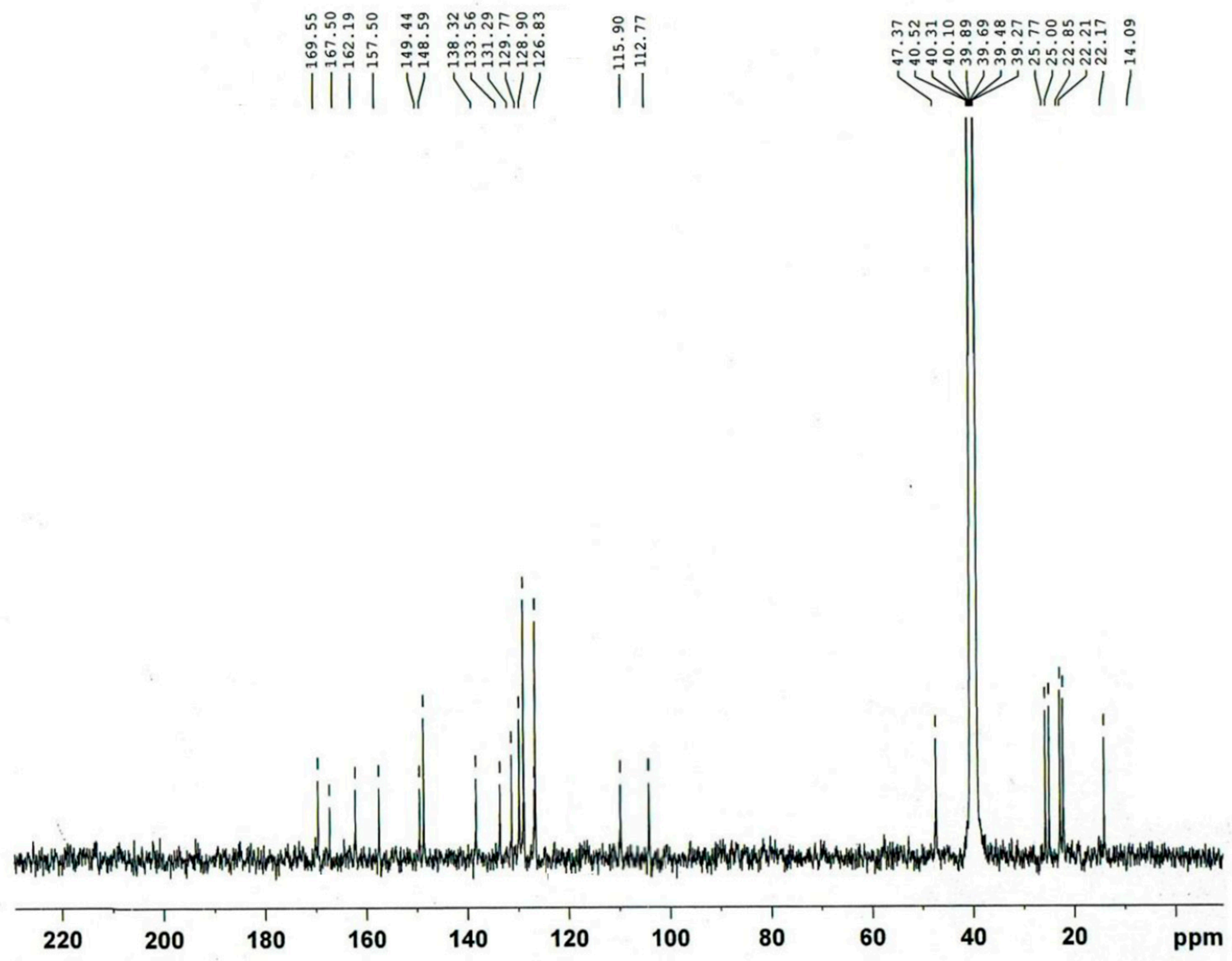
169.55
167.50
162.19
157.50
149.44
148.59
138.32
133.56
131.29
129.77
128.90
126.83
115.90
112.77
47.37
40.52
40.31
40.10
39.89
39.69
39.48
39.27
25.77
25.00
22.85
22.21
22.17
14.09
220
200
180
160
140
120
100
80
60
40
20
ppm

MASS

12I

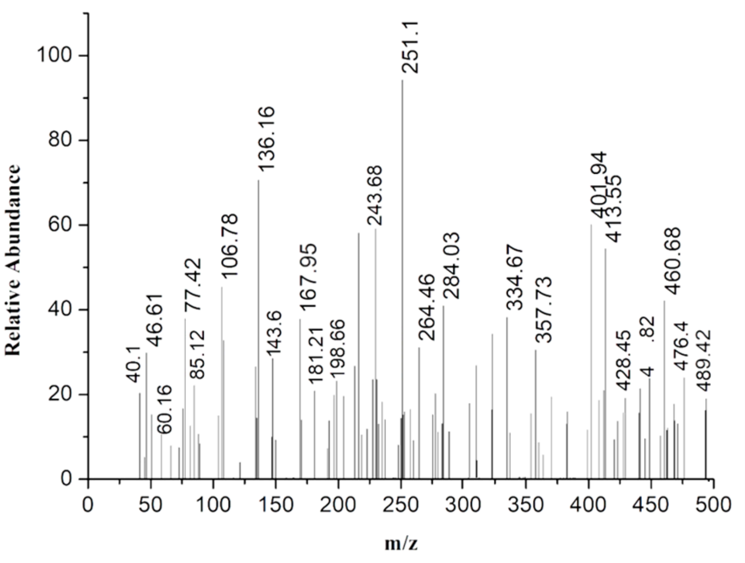

Relative Abundance
m/z
251.1
136.16
243.68
401.94
413.55
106.78
460.68
284.03
334.67
357.73
167.95
77.42
46.61
264.46
143.6
181.21
198.66
85.12
40.1
60.16
428.45
476.4
489.42

12II

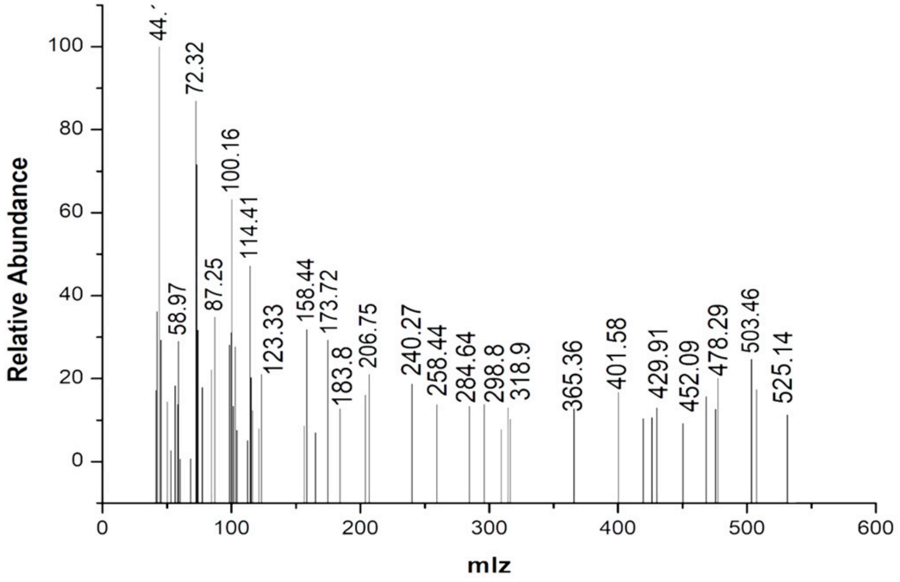
Relative Abundance
m/z
0
20
40
60
80
100
0
100
200
300
400
500
600
72.32
100.16
114.41
58.97
87.25
123.33
158.44
173.72
183.8
206.75
240.27
258.44
284.64
298.8
318.9
365.36
401.58
429.91
452.09
478.29
503.46
525.14

12III

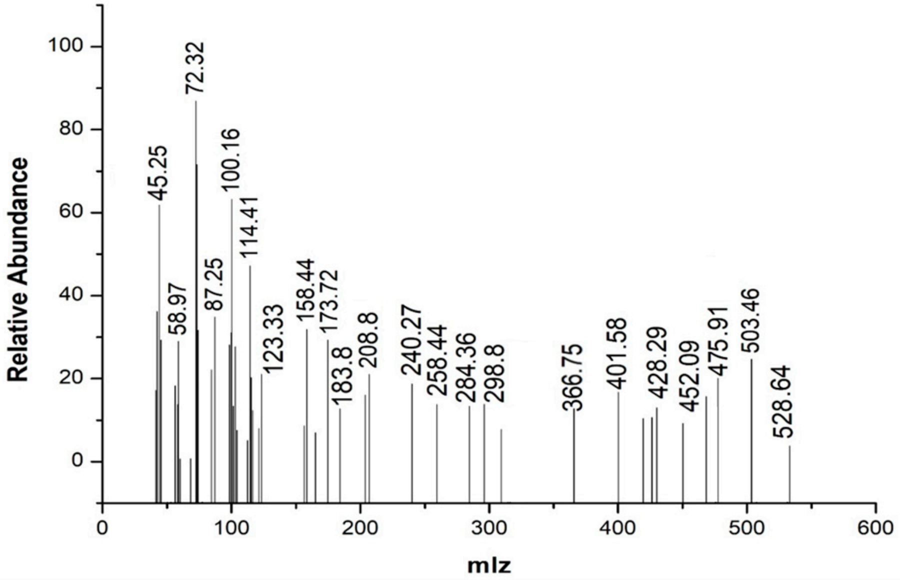

Relative Abundance
m/z
72.32
45.25
100.16
114.41
58.97
87.25
123.33
158.44
173.72
183.8
208.8
240.27
258.44
284.36
298.8
366.75
401.58
428.29
452.09
475.91
503.46
528.64
